# Association between the Cytosine Hydroxymethylation and the Expression of microRNA in Multiple Sclerosis in Polish Population

**DOI:** 10.3390/ijms241813923

**Published:** 2023-09-10

**Authors:** Justyna Basak, Danuta Piotrzkowska, Aleksandra Kucharska-Lusina, Ireneusz Majsterek

**Affiliations:** Department of Clinical Chemistry and Biochemistry, Medical University of Lodz, Mazowiecka 5, 92-215 Lodz, Polandola.kucharska@wp.pl (A.K.-L.)

**Keywords:** microRNA, multiple sclerosis, hydroxymethylation

## Abstract

Multiple sclerosis is a chronic demyelinating disorder with an unclear etiology. A key role is thought to be played by Th17 cells and microRNAs associated with Th17, such as miR-155, miR-326 and miR-223. The present study compared the methylation and hydroxymethylation levels of CpG sites within promoters of these microRNA between MS patients and controls using PBMCs and analyzed their relationship with microRNA expression. Significant intergroup differences were found between the levels of 5-hmC within the CpG-1 miR-155 promoter and CpG within the miR-326 promoter; in addition, miR-155-5p and miR-223-3p expression was elevated in MS patients. Correlation analysis showed a positive relationship between the level of 5-hmC of CpG-2 in the miR-223 promoter and miR-223-3p level. As it is possible to pharmacologically modulate the level of epigenetic modifications, our findings cast light on the etiology of MS and support the development of more effective therapies.

## 1. Introduction

Multiple sclerosis (MS), the most common demyelinating disorder of the nervous system, is the leading cause of non-traumatic disability among young adults aged 20 to 40 years. MS is a chronic disease, characterized by multifocal lesions within the myelin sheaths and axons of the central nervous system (CNS). The exact pathogenesis of MS is unclear; however, a key role is thought to be played by autoreactive immune cells directed against myelin, resulting in musculoskeletal symptoms or bladder or vision problems [1,2,3]. The causes of immune system dysfunction in MS are complex and may include environmental factors such as vitamin D deficiency or virus infection, and genetic factors such as major histocompatibility complex (MHC) genes [4,5]. In addition, many reports indicate that the pathogenic immune response may be enhanced by epigenetic modifications, particularly chromatin remodeling, DNA methylation and small non-coding RNAs [6].

MicroRNAs (miRNAs) are non-coding RNAs that play a key role in the regulation of gene expression; it is estimated that as much as 30% of human genes can be modulated by these molecules. MiRNAs are small molecules, about 20 nucleotides long, that silence gene expression by translational repression: their sequences are partially complementary to those of their targets [7]. Previous studies indicate that miRNA dysregulation plays an important role in the development of neuroinflammation and neurodegenerative disorders. Due to their wide spectrum of action, miRNAs can activate or inhibit inflammation and affect the functioning of neurons in a number of diseases, including Parkinson’s disease (PD), Alzheimer’s disease (AD), Huntington’s disease (HD), Amyotrophic lateral sclerosis (ALS), or inflammation in spinal cord injuries. In the course of neuroinflammatory diseases and injuries, miRNA activity can become deregulated, interfering with the expression of immunoglobulin receptors, neuroprotective proteins or signaling molecules, thus influencing the survival and differentiation of neurons and immune cells [8,9,10].

Although many miRNAs have been found to be deregulated in the pathogenesis of MS, those involved in the regulation of Th17 cell differentiation pathways are of particular importance. Th17 cells are subclasses of T helper (Th) CD4+ cells, characterized by the production of interleukin-17 (IL-17), responsible for cell-mediated immunity, induction of inflammation and involvement of neutrophiles; however, in the case of autoimmune disorders, Th17 are the dominant class of cells promoting a pathogenic immune response. Among the miRNAs known to be strongly associated with Th17 regulation and MS, the most important are miR-155, miR-326 and miR-223 [11].

MiR-155 is one of the most important miRNAs involved in the development of autoimmune disorders. It targets numerous genes related to inflammation, including inositol phosphatase SHIP1 regulating B cell activation [12], Dnaja1, Dnajb2 and Ets-1, negative regulators of Th17 [13,14], as well as FORKHEAD BOX 3 (FOXO3), which is responsible for regulating Th1 differentiation [15]. Elevated levels of mir-155 have been identified multiple times in the blood cells, active brain lesions and resident brain cells of MS patients [16,17]. The overexpression of miR-155 is also associated with violation of the blood–brain barrier (BBB); the activation of microglia and greater severity of MS symptoms and may significantly affect prognosis. Studies have also shown that knockdown of the miR-155 gene in an animal model of MS (EAE, experimental autoimmune encephalomyelitis) contributes to a decrease in Th1 and Th17 cells and a mild course of disease in mice; in addition, a low expression of miR-155-3p increases the myelination process and decreases microglia activation. Therefore, the modulation of miR-155 expression may be of immense importance for the treatment of MS [16,18,19].

MiR-223 and miR-326 may play a similar role to miR-155 in autoimmunity. As in the case of miR-155, a high expression of miR-223 and miR-326 is associated with exacerbation of symptoms in EAE [20,21]. Moreover, the level of miR-223 is elevated in regulatory T cells (Tregs) and whole blood, as well as in CD4+ T cells during the relapsing phase in patients with relapsing–remitting MS (RRMS) [22,23,24]. Mir-223 also plays a vital role in the regulation of hematopoietic differentiation and is recognized as one of the most important miRNAs involved in the regulation of the functioning of the immune system and the inflammatory response, which targets genes associated with the regulation of CD4+ T cells, such as STAT1 and FOXO3 [15,25,26]. MiR-326 is also a prominent regulator of CD4+, which like miR-155 is involved in silencing Ets-1, and high expression of this miRNA is also correlated with the phase of RRMS and acute course of disease [21,27]. In addition, miR-326 affects the IL-23-dependent Th17 cell regulatory pathway via disintegrin metalloprotease 17 (ADAM17), ectodomain of membrane interleukin (IL)-23R (mIL-23R) [28].

Despite the large amount of data on the role of these miRNAs in MS, the reason for their deregulation is not fully understood. A growing body of evidence indicates that epigenetic modifications, primarily DNA methylation, may be responsible for changes in the expression of many miRNAs; however, previous miRNA epigenetics research has focused on methylation in cancer [29], but few analogous analyses have been performed for autoimmune and neurological diseases such as MS. Moreover, DNA hydroxymethylation is a poorly understood epigenetic modification and hence requires deeper analysis. Current studies show that hydroxymethylated cytosine (5-hmC) residues, which are formed by the conversion of 5-methylcytosine (5-mC) with the participation of TET (ten-eleven translocation) proteins, are common in neurons and may play a role in regulating gene activity [30,31]. However, there are few examples of data on the role of 5-hmC tags in the regulation of miRNA gene expression. Nevertheless, an animal study is promising and indicates that miR-365-3p can be regulated by hydroxymethylation of the promoter sequence; it suggests that hydroxymethylation and DNA methylation can significantly influence the expression of mature miRNAs [32].

The present study compared the methylation and hydroxymethylation levels of the miR-155, miR-223 and miR-326 promoters between MS patients and controls using peripheral blood mononuclear cells (PBMCs). It also analyzed the relationship between these modifications with the expression of mature miRNAs. We believe that our findings can significantly contribute to the development of better MS therapeutics and provide greater insight into the regulation of miRNA expression in autoimmune and neurodegenerative disorders.

## 2. Results

**Figure 1:**
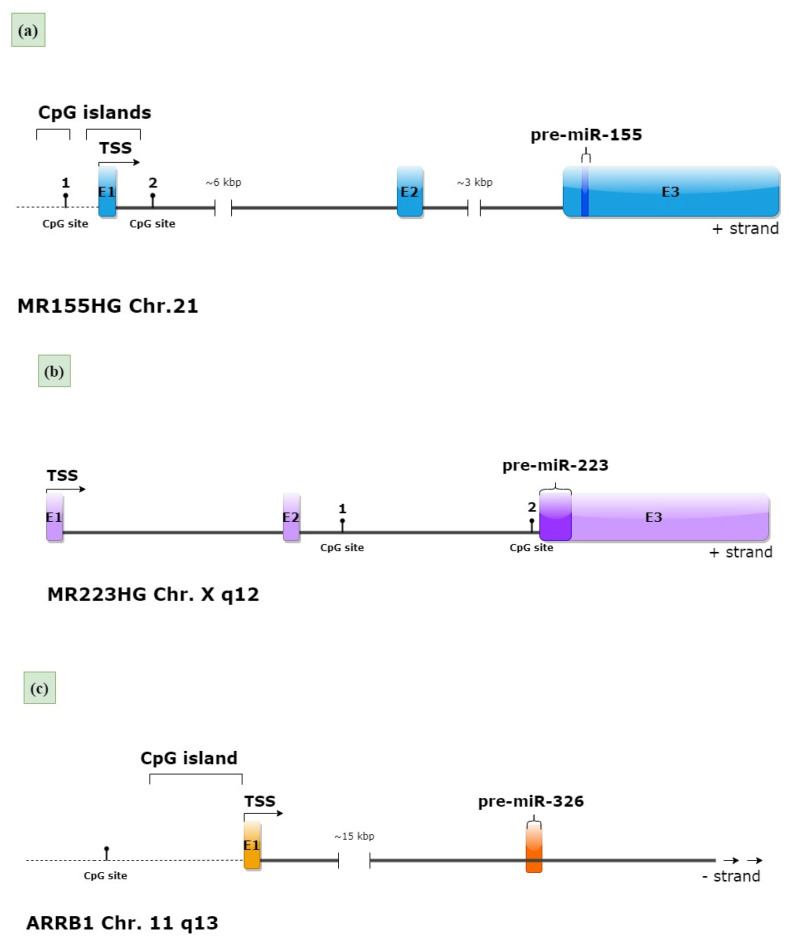
The structure of the miRNA host genes and the location of pre-miRNA sequences with selected CpG sites. Black dots and numbers indicate selected CpG sites. (**a**) The miR-155 gene is located within exon 3 of the *MIR155HG* host gene and is regulated by the *MIR155HG* promoter [33]. Upstream (−400 bp and −80 bp) of the transcription site start (TSS) are two CpG islands, according to MethPrimer software (https://www.urogene.org/methprimer/ accessed on 7 November 2022); (**b**) the miR-223 gene is located within exon 3 of the *MIR223HG* host gene. The region upstream of exon 3 of the *MIR223HG* gene is believed to regulate miR-223 expression [34]. (**c**) The miR-326 gene is located within the first intron of the *ARRB1* gene (arrestin beta 1) and is regulated by the *ARRB1* promoter (the figure shows a fragment of the *ARRB1* gene, arrows represent the continuity of the gene) [35]. MethPrimer (https://www.urogene.org/methprimer/ accessed on 7 November 2022) indicates CpG-rich region −600 bp upstream of the transcription site start (TSS).

The locations of the promoter sequences of the miR-155, miR-223 and miR-326 genes were identified in silico. Based on the obtained data and previous literature, regions rich in CpG sites were selected, including two CpG sites for the miR-155 and miR-223 promoters and one CpG site for the miR-326 promoter. The structure of the miRNA host genes and the location of pre-miR-155, pre-miR-223 and pre-miR-326 sequences, as well as the selected CpG sites, are presented in Figure 1.

### 2.1. Methylation and Hydroxymethylation Levels of miR-155, miR-223 and miR-326 CpG Sites

**Figure 2:**
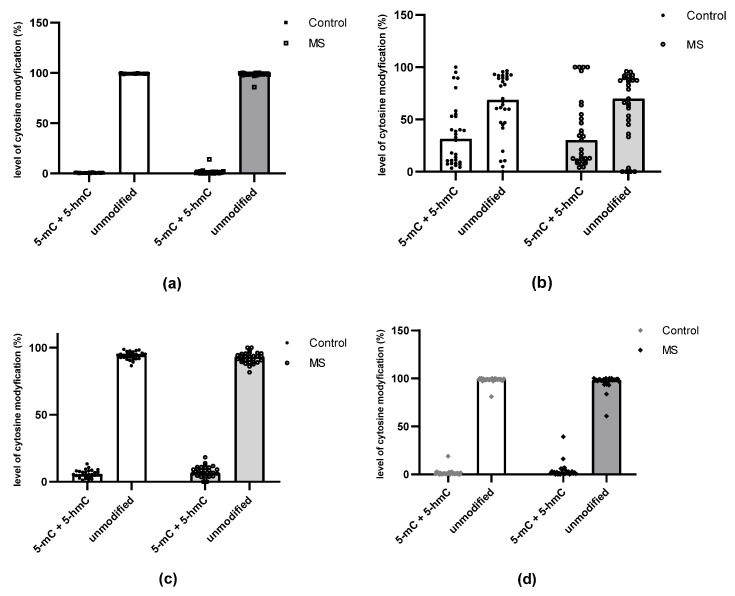
Comparison of the total level of cytosine modifications (5-mC and 5-hmC) with the level of unmodified cytosines within the selected CpG site in MS patients and controls. (**a**) CpG-1 within the miR-155 promoter. (**b**) CpG-1 within the miR-223 promoter. (**c**) CpG-2 within the miR-223 promoter. (**d**) CpG-2 within the miR-326 promoter. Data are presented as individual points with median.

The analyzed CpG sites indicated varying degrees of cytosine modification (Figure 2). A particularly high percentage of modified cytosine was observed in the CpG-1 of the miR-223 promoter region: the percentage of modified cytosines also demonstrated high individual variability in CpG-1, both in the control and the study group; however, no significant differences between these groups were found. The CpG-2 of this promoter was modified to a much lesser extent, and both groups exhibited similar percentages of modifications.

**Figure 3:**
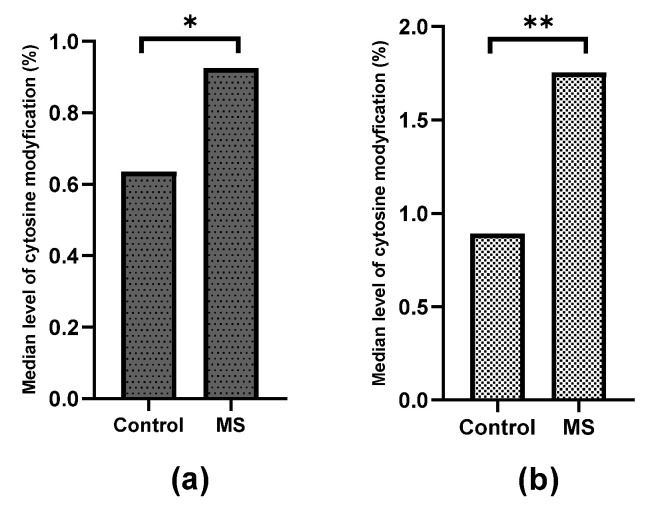
Comparison of the level of total cytosine modifications (5-hmC + 5-mC) between patients and controls. (**a**) CpG-1 within miR-155 promoter. (**b**) CpG-2 within miR-326 promoter. The groups of data were compared using the Mann–Whitney U-test (* indicate statistical significance at a *p* < 0.05, ** indicate statistical significance at a *p* < 0.01).

Within the miR-155 promoter, the selected CpG-2 site was not modified in either the control group or in the study group. Although CpG-1 within the miR-155 promoter and CpG within the miR-326 promoter were modified to a very small extent in both MS and controls, significant differences in the degree of modification were found between patients and controls (*p* = 0.01263 and *p* = 0.00891, respectively, for miR-155 and miR-326) (Figure 3).

**Figure 4:**
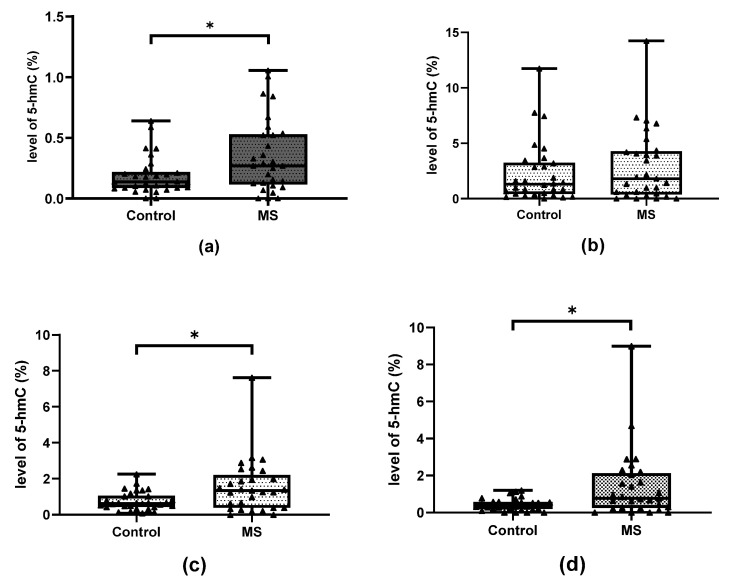
Comparison of the level of 5-hmC in the group of patients and the control group (**a**) CpG-1 within miR-155 promoter. (**b**) CpG-1 within miR-223 promoter. (**c**) CpG-2 within miR-223 promoter. (**d**) CpG-2 within miR-326 promoter. Data are shown as individual points with median, minimum and maximum values marked. The groups of data were compared using the Mann–Whitney U-test (* indicate statistical significance at a *p* < 0.05).

**Figure 5:**
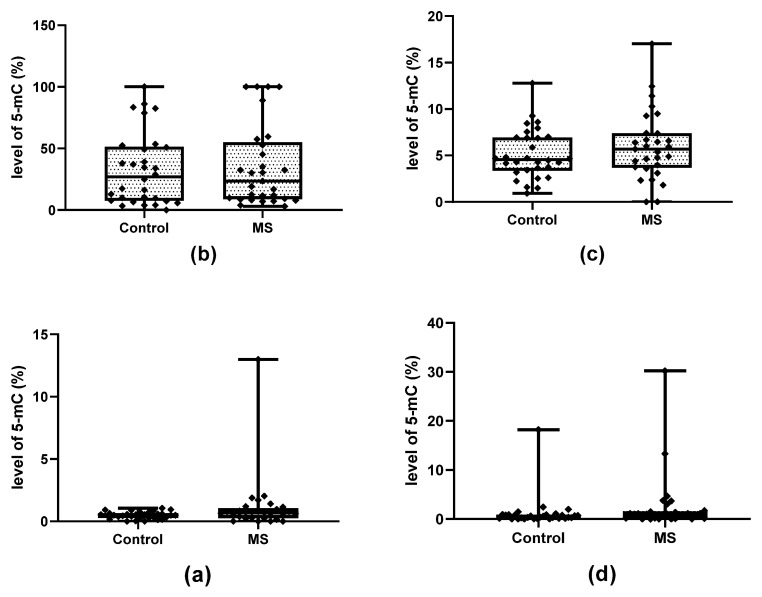
Comparison of the level of 5-mC in the group of patients and the control group (**a**) CpG-1 within the miR-155 promoter. (**b**) CpG-1 within the miR-223 promoter. (**c**) CpG-2 within the miR-223 promoter. (**d**) CpG-2 within the miR-326 promoter. Data are shown as individual points with median, minimum and maximum values marked. The groups of data were compared using the Mann–Whitney U-test.

Significant differences in the level of hydroxymethylation were also found between patients and controls in the case of CpG-2 miR-223, CpG-1 miR-155 and CpG miR-326 (Figure 4) (*p* = 0.03442 for miR-155, *p* = 0.02435 for miR-223 and *p* = 0.01210 for miR-326); however, no differences in methylation level were found in any case (Figure 5).

### 2.2. Expression Levels of miR-155-5p, miR-223-3p, miR-223-5p and miR-326

**Figure 6:**
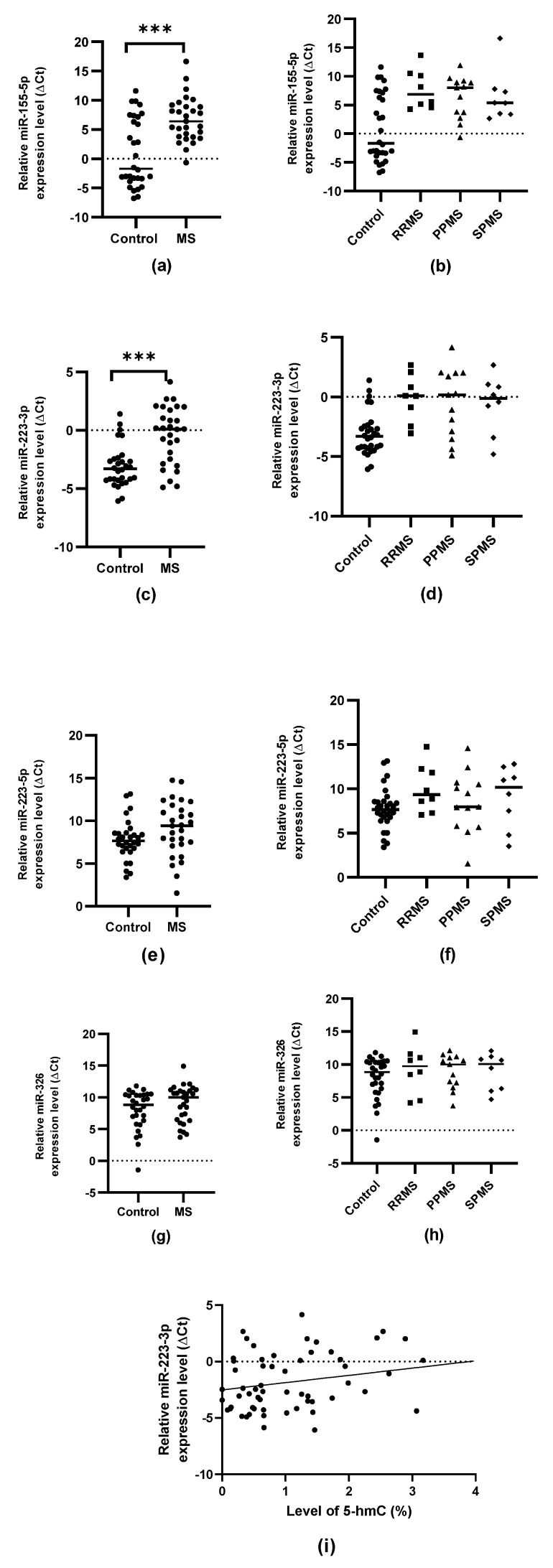
Expression of selected miRNAs and correlation with 5-hmC level in PBMCs from MS patients and controls. Relative ΔCt expression levels are shown for: (**a**) miR-155-5p, (**b**) miR-155-5p in in controls and MS patients (RMMS [*N* = 8], PPMS [*N* = 13] and SPMS [*N* = 8]), (**c**) miR-223-3p, (**d**) miR-223-3p in controls and MS patients (RMMS [*N* = 8], PPMS [*N* = 13] and SPMS [*N* = 8]), (**e**) miR-223-5p, (**f**) miR-223-5p in controls and MS patients (RMMS [*N* = 8], PPMS [*N* = 13] and SPMS [*N* = 8]), (**g**) miR-326 (**h**) miR-326 in controls and MS patients (RMMS [*N* = 8], PPMS [*N* = 13] and SPMS [*N* = 8]); (**i**) Spearman correlation scatterplot for the 5-hmC level within the CpG-2 of the miR-223 promoter and the relative expression of miR-223-3p for the overall cohort (*N* = 59). The groups of data were compared using the Mann–Whitney U-test. (*** indicate statistical significance at *p* < 0.001).

The expression of miR-155-5p and miR-223-3p in PBMCs was significantly elevated in MS patients compared to controls (*p* = 0.00045 and *p* = 0.00012, respectively); however, no significant differences in miR-223-5p or miR-326 were found. In addition, no significant differences in expression were found between patients with RRMS, PPMS and SPMS. A slight positive relationship was found (Spearman’s rank correlation coefficient R = 0.26468, *p* = 0.04278) between the level of 5-hmC within the CpG-2 of the miR-223 promoter and the level of expression of miR-223-3p in the study group (*N* = 59) (Figure 6).

## 3. Discussion

Our data provide new insights into the role of hydroxymethylation in the regulation of miRNA involved in the pathogenesis of MS. Hydroxymethylation is a relatively poorly understood epigenetic marker, which initially was thought to be an intermediate of DNA demethylation; however, further research has shown that it may be involved in the regulation of genomic structure and function. Moreover, it has been proven that 5-hmC is particularly important in the proper functioning of nervous system and its deregulation was also associated with several human diseases, including solid tumors and leukemia [36]. Previous studies have also shown that global hydroxymethylation may be impaired during MS due to reduced expression of TET2 [37], and that the reduction in hydroxymethylation and TET levels in the spinal cord is associated with the induction of EAE in a mouse model [38]. However, it has not yet been determined whether hydroxymethylation may be involved in the regulation of miRNAs related to autoimmunity in MS.

The epigenetic factors modifying the expression of deregulated miRNAs are of particular interest due to their potential for modulation by enzyme inhibitors, which are already being evaluated as candidates in new anticancer strategies [39]. Therefore, a better understanding of the epigenetic mechanisms in miRNA regulation may lead to the development of therapies targeting these processes, which could improve the existing methods of MS treatment.

Our findings are the first to indicate a relationship between the CpG 5-hmC levels of the miR-155, miR-223 and miR-326 promotors in MS patients. Although the 5-hmC level in each analyzed CpG site is relatively small, all selected miRNA demonstrated 5-hmC increase in MS patients. Only one of the selected CpG sites within the miR-223 promoter did not exhibit any difference in 5-hmC level between the studied groups; however, it is important to note that different sites located in CpG islands can be modified in various ways. The present study was restricted to certain promoter sequences and specific CpG islands, as well as a limited range of sites to allow cleavage by restriction enzymes and the optimization of the method, primarily the selection of primers. In addition, it is highly probable that miRNA expression is modulated by the interaction of many such modifications located both in promoters and in enhancer sequences.

Nevertheless, the identified differences between the group of MS patients and controls indicate that the selected CpG sites indeed have regulatory potential. Interestingly, although methylation is considered to be the key regulatory modification of cytosine, no differences in the level of 5-mC were found in the analyzed sites; however, the total level of 5-hmC and 5-mC was greater in the MS patient group compared to controls, which may indicate that the 5-hmC markers may play a more significant role in the modulation of miRNA expression than was previously assumed. Pan et al. identified an association between the elevated 5-hmC level and an increase in miR-365-3p transcription in mice [32], suggesting that a similar regulation of miRNA expression may occur in humans.

To better assess the impact of 5-hmC on the level of mature miRNAs, the study also analyzed the expression of miR-155-5p, miR-223-3p and 5p and miR-326; the findings partially confirmed earlier reports that these miRNAs were of elevated in the PBMCs of MS patients [40,41]. More specifically, an increased expression of miR-155-5p and miR-223-3p was noted in all three types of MS, but no significant differences were found between these groups. However, due to the small samples of patients with RRMS, PPMS and SPMS, it cannot be ruled out that the level of expression of the studied miRNAs may depend on the type and course of the disease. Indeed, earlier studies on extracellular miRNAs show that miR-326-5p showed significantly higher expression in RRMS patients compared to SPMS, and hence may serve as a biomarker differentiating between the two conditions [42]. However, our present findings were unable to confirm that miR-326 expression was significantly impaired in MS patients, although a slight upregulation was noted compared to control. This may also be due to the limitations of our experiment, resulting from the small number of patients enrolled and individual differences between ethnic groups.

It was not possible to confirm whether the miR-223-5p variant is significantly elevated in MS; like miR-326, miR-223-5p expression was slightly elevated in MS patients, but not significantly so. However, our results are particularly interesting, because previous studies have rarely analyzed the expression of both variants of this miRNA. Our results suggest that the -3p variant may have a more significant role during MS than the -5p variant. This is of interest because miRNA variants in the -5p arm are more often considered canonical. Studies also indicate that switching between the -5p and -3p arms may allow miRNA activity to be regulated since the different variants of the same miRNA can target different genes, and miR-5p and miR-3p expression varies dynamically; as such, individual variants may be expressed differently in response to the functional needs of the organism [43]. Thus, further research is also required to understand how these mechanisms may play a role in pathological conditions.

The analysis of the correlation between the level of the studied epigenetic modifications and the expression of miR-155-5p, miR-223-3p, miR-223-5p and miR-326 showed a weak positive relationship between the level of 5-hmC at the miR-223 promoter CpG-2 site and the expression of the miR-223-3p variant. Interestingly, no such relationship was noted for the -5p variant, which may also suggest that the miR-223-3p variant is preferable. The fact that a positive relationship exists between the level of 5-hmC and the intensity of expression of the gene regulated by hydroxymethylation is consistent with previous reports. Lin et al. report that regions rich in 5-hmC tags are transcriptionally active and that intragenic 5-hmC enrichment is associated with higher levels of expression [44]. Similar conclusions were reached by Stroud et al., who additionally suggest that hydroxymethylated sites may mediate the binding of transcription factors and enhancers [45]. Moreover, Valinluck et al. indicate that 5-hmC modifications can inhibit the binding of methyl-CpG binding protein 2 (MeCP2) [46]. MeCP2 is a protein with various functions, strongly involved in the regulation of transcription and chromatin organization; in addition, MeCP2 plays an important role in modulating the accessibility of primary transcripts to processing enzymes, resulting in an increase in mature miRNA level [47,48]. Therefore, the exact relationship between hydroxymethylation and miRNA expression requires more depth examination.

Our findings provide greater insights into the mechanisms regulating miRNA expression related to autoimmunity and neurodegeneration; however, due to the limitations of our research, further investigation is necessary to fully elucidate the importance of these modifications. It is important to note that our study was conducted in a small group of Caucasian patients, and as such, it is possible that these results may not be reflected in other ethnic groups, other types of MS or on a larger scale. It should also be emphasized that changes in cytosine modifications are a dynamic process that can be influenced by the environment [49]; therefore, they should be analyzed in a broader context, considering the diet, therapy and lifestyle of the patients. Moreover, as the degree of methylation is largely dependent on the stage of development of the organism, the modifications examined in the present study may be significantly influenced by the age of patients and the aging process itself [50]. Also, due to the small size of the research group, our study provides little data on the impact of the duration of the disease on epigenetic mechanisms.

It is therefore necessary to confirm our results in a larger and more diverse study group. In addition, an analysis of the global hydroxymethylation and methylation levels of the genes of interest would be particularly valuable, as this will give a broader picture of miRNA regulation through cytosine modifications. Although our findings do not confirm any relationship between methylation level and the expression of the selected miRNA, it is not possible to completely rule out their participation, due to the limitations in the selection of CpG sites for analysis and the small study group. Nevertheless, our findings shed new light on the mechanisms of miRNA regulation, and due to the possibility of pharmacological modulation of the level of epigenetic modifications, they may contribute to the development of more effective MS therapies.

## 4. Materials and Methods

In total, 29 patients with MS (Table 1) diagnosed according to the McDonald criteria (version 2017) and 30 healthy people (Table 2) from the Vadimed Medical Center in Krakow, Poland were recruited for the study. The study group comprised individuals between 18 and 70 years; all people outside this age range were excluded. The control group was matched to the study group with regard to age and sex. The exclusion criteria also included people suffering from mental illnesses, cancer and other neurological and autoinflammatory diseases, as well as MS patients with disorders or symptoms that prevented or hindered verbal contact. The research was approved by the Bioethics Committee of the Medical University of Lodz, and all participants gave their written informed consent to attend the study. Before starting the experiments, all participants underwent medical examinations.

### 4.1. PBMCs DNA and RNA Purification

Total DNA was extracted from whole blood samples from each study subject using a commercially available Blood Mini (A&A Biotechnology, Gdansk, Poland) kit for genomic DNA extraction according to the manufacturer’s protocol. Total RNA was extracted from whole blood using a RiboPure™ RNA Purification Kit (Invitrogen™, ThermoFisher Scientific, Waltham, MA, USA). The concentration and quality of the purified DNA and RNA were assessed by spectrophotometric measurement of samples at 260 and 280 nm using a SYNERGY HTX microplate reader (BioTek Instruments, Inc, Winooski, VT, USA).

### 4.2. Analysis of the Level of Methylation and Hydroxymethylation

The level of 5-mC and 5-hmC was assessed using Thermo Scientific EpiJET 5-hmC and 5-mC Analysis Kit (Thermo Scientific™, ThermoFisher Scientific, Waltham, MA, USA). According to the manufacturer’s instructions, each glucosylation reaction used 1 µg of DNA. Digestion with the restriction enzymes T4 BGT, Epi MspI and Epi HpaII was then performed according to the manufacturer’s protocol. Pre-prepared samples were subsequently analyzed using qPCR with specific primers flanking selected CpG sites (Table S1) and Maxima SYBR Green/ROX qPCR Master Mix (Thermo Scientific™, ThermoFisher Scientific, Waltham, MA, USA). The qPCR was performed using a CFX Connect Real-Time PCR Detection System (Bio-Rad, Hercules, CA, USA). For the qPCR reaction, 1 µL of digested DNA was used as a template, 10 µL of PCR Master mix, 0.3 µM of forward and reverse primers and the appropriate amount of nuclease-free water. The total volume of the reaction mixture was 20 µL. Each trial was performed in two independent replicates. The qPCR conditions are shown in Table S2.

### 4.3. Analysis miRNA Expression

The level of miRNA expression was assessed by RT-qPCR using commercially available kit TaqMan™ MicroRNA Reverse Transcription kit (Applied Biosystems™, ThermoFisher Scientific, Waltham, MA, USA) and probes specific to selected miRNAs TaqMan™ MicroRNA Assay (Applied Biosystems™, ThermoFisher Scientific, Waltham, MA, USA), as well as TaqMan™ Universal Master Mix II, no UNG (Applied Biosystems™, ThermoFisher Scientific, Waltham, MA, USA). The RT-PCR reaction was performed using a T100 Thermal Cycler (Bio-Rad, Hercules, CA, USA). According to the manufacturer’s protocol, 300 ng of total RNA was used per 15 µL RT reaction. The qPCR was performed using a CFX Connect Real-Time PCR Detection System (Bio-Rad, Hercules, CA, USA). For the qPCR reaction, 1.33 µL of cDNA was used as a template, 10 µL of PCR Master mix, 1 µL TaqMan™ Small RNA Assay and 7.67 µL of nuclease-free water. The total volume of the reaction mixture was 20 µL. Each trial was performed in two independent replicates. The RT-qPCR conditions are shown in Table S3 hsa-miR-191-5p and hsa-miR-103a-3-p were used as reference genes.

## Figures and Tables

**Table 1:** Clinical characteristics of the study group, consisting of eight patients with relapsing-remitting MS (RRMS), thirteen patients with primary progressive MS (PPMS) and eight patients with secondary progressive MS (SPMS).

Characteristic of Study Group	Total
Number of MS patients	29
RRMS	8
PPMS	13
SPMS	8
Females/males	22/7
Age (years, $\bar{X}$ ± s)	47.9 ± 14.1
Disease duration (years, $\bar{X}$ ± s)	5.7 ± 2.6
Expanded disability status scale (EDSS) (scale 1–10) ^1^	5.4 ± 0.8
0–5.5	16 (55.2%)
6–6.5	13 (44.8%)

^1^ 0–5.5—no or little impairment to walking; 6–6.5—requires one or two walking aids, >7—wheelchair mobility or confined to bed.

**Table 2:** Characteristics of the control group.

Characteristics of Control Group	Total
Number	30
Age (years, $\bar{X}$ ± s)	55.6 ± 17.7
Females/males	13/17

## Data Availability

Not applicable.

## Supplementary Materials

The following supporting information can be downloaded at: https://www.mdpi.com/article/10.3390/ijms241813923/s1.
