# Supplementary material for: Association between the Cytosine Hydroxymethylation and the Expression of microRNA in Multiple Sclerosis in Polish Population
Source: Int J Mol Sci. 2023 Sep 10;24(18):13923. doi: 10.3390/ijms241813923 (PMC10531266; doi:10.3390/ijms241813923)
Supplement: Supplementary file 1 [file ijms-24-13923-s001.zip › ijms-2577708-supplementary.pdf]

Table S1. Primer sequences for methylation and hydroxymethylation analysis.

miRNA	Forward Primer	Reverse Primer
miR-155	GCAATGACCCACGAGAAAGGG	GCCTGTTCTTGGAACCTACAG
	GACTCAGTGGTGTCGCCTC	CAGGTACGTCCCCAACCATC
miR-223	GGGTGTCACTCGGGCTTTAC	TTCCTGGGAGATGTGACCCT
	AGACAAGGCTGAATCCCTCTG	CCAGAAATGGTAAACTGCCCC
miR-326	AAGCTTAGTGCACAGGGTCA	GCGATAAAGGAAGAGGGCAGA

Table S2. The qPCR conditions for methylation and hydroxymethylation analysis.

Step	Temperature	Time	Number of cycles
Initial denaturation	95°C	10 min	—
Denaturation	95°C	15 s	40
Annealing	60°C	30 s	
Extension	72°C	30 s	

Table S3. The qPCR conditions for miRNA expression analysis. .

QRT reaction			
**Step**	**Temperature**	**Time**	
Reverse transcription	16°C	30 min	
	42°C	30 min	
Stop reaction	85°C	5 min	
**qPCR**			
**Step**	**Temperature**	**Time**	**Number of cycles**
Enzyme activation	95°C	10 min	—
Denaturation	95°C	15 s	40
Annealing and extension	60°C	60 s	
